# Is Standardization Transferable? Initial Experience of Urethral Surgery at the University Hospital Frankfurt

**DOI:** 10.3389/fsurg.2020.600090

**Published:** 2020-12-14

**Authors:** Mike Wenzel, Marieke J. Krimphove, Benedikt Lauer, Benedikt Hoeh, Matthias J. Müller, Philipp Mandel, Andreas Becker, Malte W. Vetterlein, Stefan C. Mueller, Roland Dahlem, Margit Fisch, Felix K.-H. Chun, Luis A. Kluth

**Affiliations:** ^1^Department of Urology, University Hospital Frankfurt, Frankfurt, Germany; ^2^Department of Urology, University Hospital Hamburg-Eppendorf, Hamburg, Germany

**Keywords:** urethral stricture, urethroplasty, patient-reported outcome measure, PROM, QOL, buccal mucosal graft urethroplasty

## Abstract

**Background:** Since January 2018 performance of urethroplasties is done on regular basis at the University Hospital Frankfurt (UKF). We aimed to implement and transfer an institutional standardized perioperative algorithm for urethral surgery (established at the University Hospital Hamburg-Eppendorf—UKE) using a validated Urethral Stricture Surgery Patient-Reported Outcome Measure (USS-PROM) in patients undergoing urethroplasty at UKF.

**Materials and Methods:** We retrospectively analyzed all patients who underwent urethroplasty for urethral stricture disease between January 2018 and January 2020 at UKF. All patients were offered to revisit for clinical follow-up (FU) and completion of USS-PROM. Primary end point was stricture recurrence-free survival (RFS). Secondary endpoints were functional outcomes, quality of life (QoL), and patient satisfaction.

**Results:** In total, 50 patients underwent urethroplasty and 74 and 24% had a history of previous urethrotomy or urethroplasty, respectively. A buccal mucosal graft urethroplasty was performed in 86% (*n* = 43). After patient's exclusion due to lost of FU, FU <3 months, and/or a pending second stage procedure, 40 patients were eligible for final analysis. At median FU of 10 months (interquartile-range 5.0–18.0), RFS was 83%. After successful voiding trial, the postoperative median Qmax significantly improved (24.0 vs. 7.0 mL/s; *p* < 0.01). Conversely, median residual urine decreased significantly (78 vs. 10 mL; *p* < 0.01). Overall, 95% of patients stated that QoL improved and 90% were satisfied by the surgical outcome.

**Conclusions:** We demonstrated a successful implementation and transfer of an institutional standardized perioperative algorithm for urethral surgery from one location (UKE) to another (UKF). In our short-term FU, urethroplasty showed excellent RFS, low complication rates, good functional results, improvement of QoL and high patient satisfaction. PROMs allow an objective comparison between different centers.

## Introduction

Urethral surgery is a complex urological field (1–3). In contrast to other, no less complex urological procedures (e.g., HoLEP or radical prostatectomy), the intraoperative course is often unpredictable (4, 5). Despite various attempts to preoperatively evaluate the complexity of the procedure—either clinically (e.g., based on stricture length and localization) or by the use of prognostic models (6)—the definitive surgical procedure and technique usually is decided intraoperatively. The local findings of the tissue and the extent of the spongiofibrosis determine which technique will be performed and whether the procedure will be one- or even multi-staged. Based on these considerations, urethral surgeons require the ability to perform the entire surgical spectrum of urethral surgery (7).

Due to its complexity, urethroplasties are mainly performed at urethral reconstructive referral centers. Since it has been shown in other urological entities that both standardization and operative volume are significantly associated with the postoperative outcome (8), the rarity and complexity of urethroplasty makes it crucial to establish standardization as an error prevention strategy and for quality control. Given the lack of European urethroplasty guidelines on perioperative management for diagnostic and treatment of urethral strictures, institutional standards are the basis for evidence based-medicine, especially in rare diseases and surgeries. Recently, Vetterlein et al. demonstrated that a standardized voiding trial for urethral stricture patients who underwent buccal mucosa graft urethroplasty (BMGU), can improve outcomes by identifying those patients who are at high risk for early stricture recurrence (9). However, there is a lack of evidence in the contemporary literature addressing standardization in urethral surgery.

Since January 2018, urethral surgeries have been performed regularly at the Frankfurt University Medical Center (UKF). The surgeons were trained in urethral surgery at the University Hospital Hamburg-Eppendorf (UKE) for nearly a decade. The aim of the study was to implement and transfer an institutional standardized perioperative algorithm for diagnostic and treatment of urethral surgery from one location (UKE) to another (UKF). Therefore, we assessed surgical outcome, complications, functional outcomes and quality of life in patients undergoing urethral surgery for urethral stricture disease by using a validated Urethral Stricture Surgery Patient-Reported Outcome Measure (USS-PROM) (10, 11).

## Materials and Methods

### Study Cohort

This study was approved by the ethic committee of UKF (82/19). We retrospectively examined all patients in our prospective urethral stricture database who underwent urethroplasty due to urethral stricture disease at UKF from January 2018 to January 2020. Primary endpoints were stricture recurrence-free survival (RFS). Secondary endpoints were functional outcomes, complications, quality of life and patient satisfaction.

### Institutional Standardized Perioperative Algorithm

Standardization is a norming of defined processes of specific procedures (e.g., diagnostics and therapies), which needs control on regular basis by using, for example, outcome analysis. The surgeons of this study were trained in urethral surgery at the UKE for nearly a decade. An institutional standardized peri- and postoperative algorithm for diagnostic and treatment of urethral surgery established at the UKE (9, 12) was implemented at the UKF. Therefore, physicians, nurses, and surgical nurses underwent special education and training programs for the management of patients with urethral stricture disease.

### Preoperative Diagnostic Evaluation

**Figure 1:**
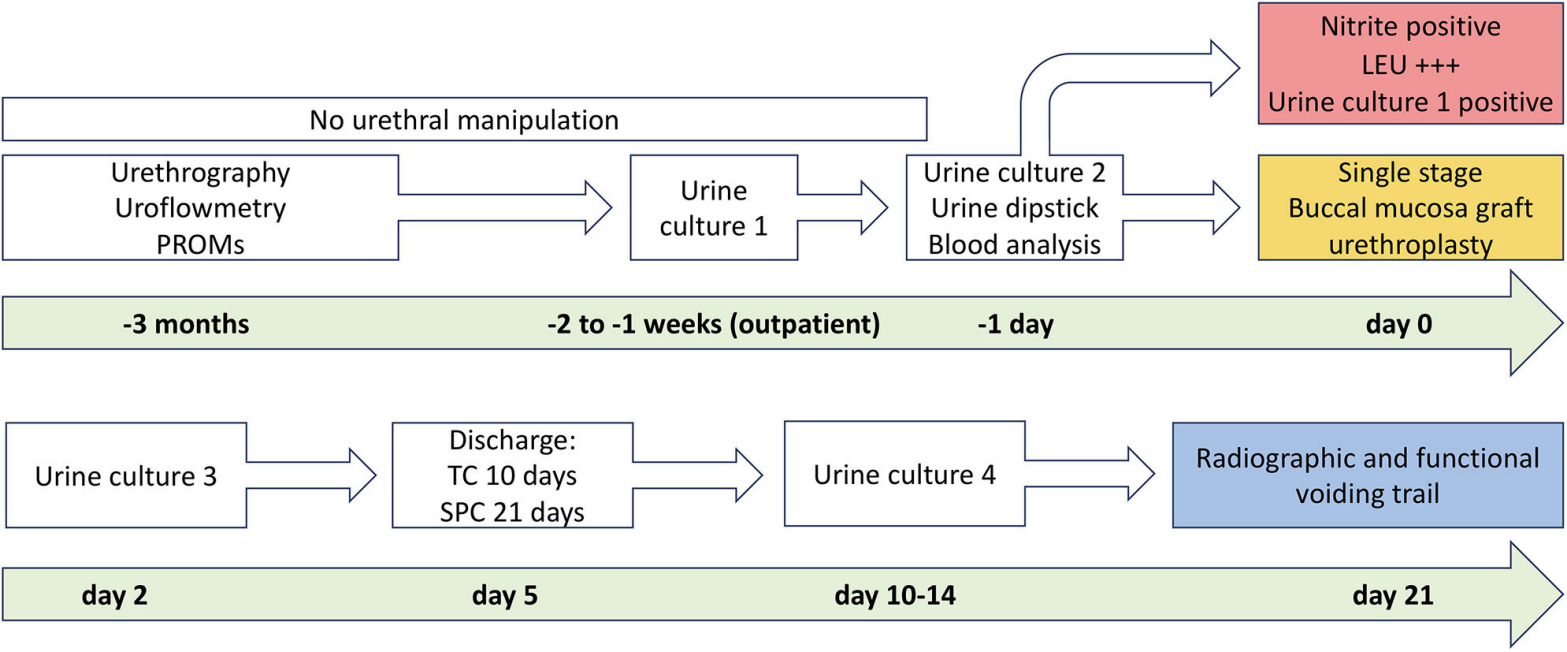
Institutional algorithm for patients undergoing buccal mucosal graft urethroplasty. The upper column of the figure shows the preoperative algorithm before and the lower column after surgery until planned voiding trial. PROM, patient-reported outcome measure; TC, transurethral catheter; SPC, suprapubic catheter. With kind permission to use figure by M. W. Vetterlein (University Medical Center Hamburg-Eppendorf).

In urethral stricture patients, the preoperative diagnostic evaluation included a detailed medical history [e.g., previous radiation (13)], physical examination, urine analysis, uroflowmetry, sonography and residual urine measurement, and a combined retrograde urethrography (RUG) and micturition cystourethrography (MCU; Figure 1).

### Surgical Procedure

**Figure 2:**
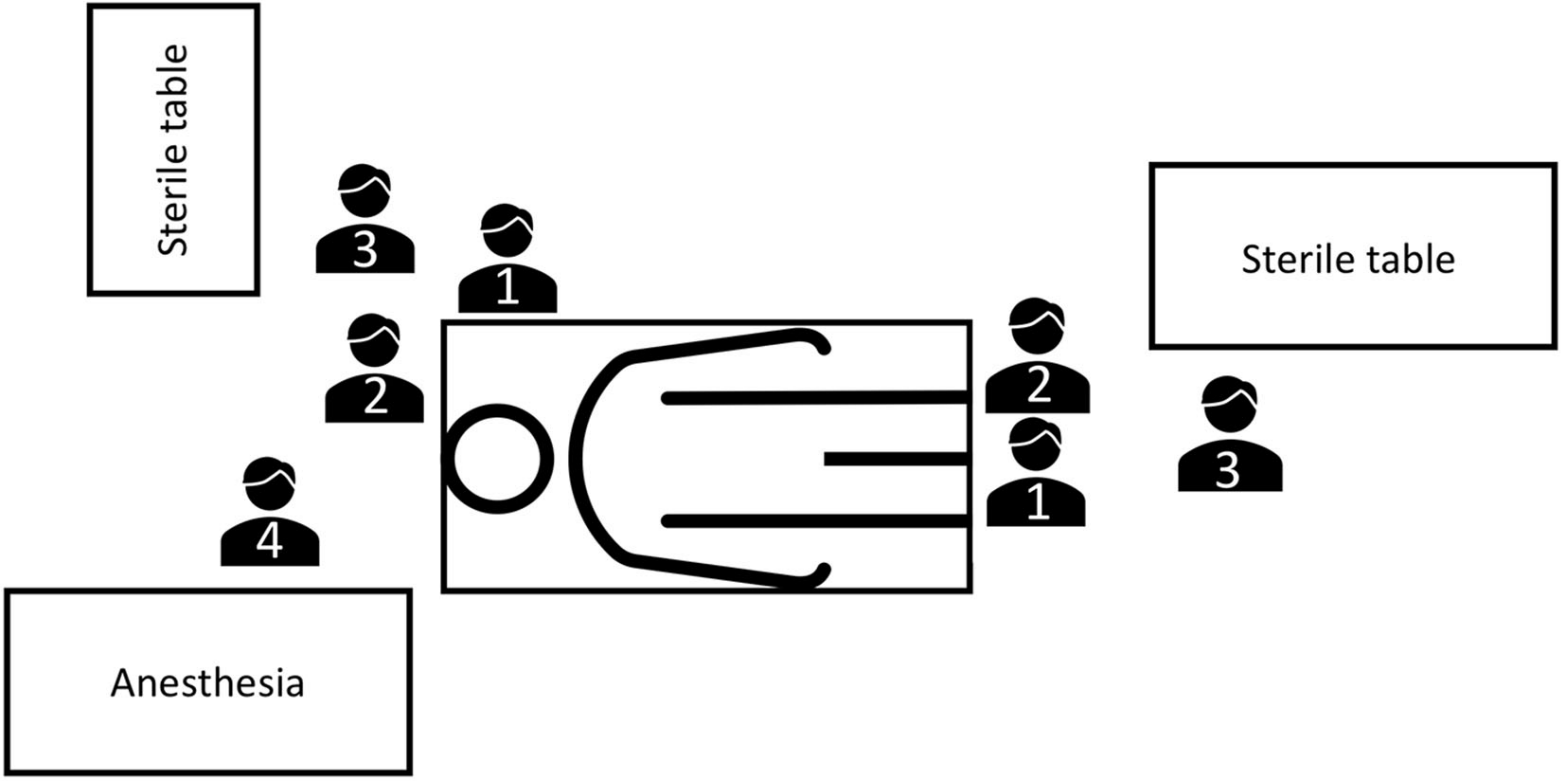
OR setting for buccal mucosa graft urethroplasties at University Hospital Frankfurt. One main surgeon and one assistant are performing the urethroplasty and harvesting the buccal mucosa graft. OR, operating room; 1, main surgeon; 2, surgeon's assistant; 3, OR nurse; 4, anesthetist.

The surgical procedure was performed by one main surgeon and one assistant (Figure 2). According to our institutional protocol, surgical technique depended on length and localization of urethral stricture, extent of spongiofibrosis and previous urethral surgeries. In short bulbar urethral strictures (≤1 cm), an excision and primary anastomosis or buccal mucosa graft urethroplasty (BMGU) was performed (14, 15). BMGU was performed as ventral onlay in any other bulbar strictures (9, 16, 17) and as dorsal inlay (5) in mid-penile strictures. In distal-penile strictures (with or without involvement of the navicular fossa), a dorsal inlay or preputial flap (4, 12) was performed, as a one or two-stage procedure. In panurethral strictures a two-stage mesh graft urethroplasty (split thickness skin graft of inner thigh) was performed (1). The decision for a one- or two-stage procedure was made intraoperatively, and all patients were informed preoperatively accordingly for a possible two-stage procedure.

### Peri- und Postoperative Management

Patients undergoing BMGU and excision and primary anastomosis were discharged on the fifth and seventh postoperative day, respectively. For initial BMGUs (no previous open reconstruction), transurethral (10 days) plus suprapubic catheterization (21 days) was performed (9, 12), while transurethral catheterization only (21 days) was performed in the re-operative setting (9, 18). After the first session of a two-stage procedure, patients were trained for urethral packing of their proximal urethra after each micturition (1, 19). The final closure or tubularization of the urethra was performed not earlier than 3 months after the first session.

### Radiographic and Functional Voiding Trial

**Figure 3:**
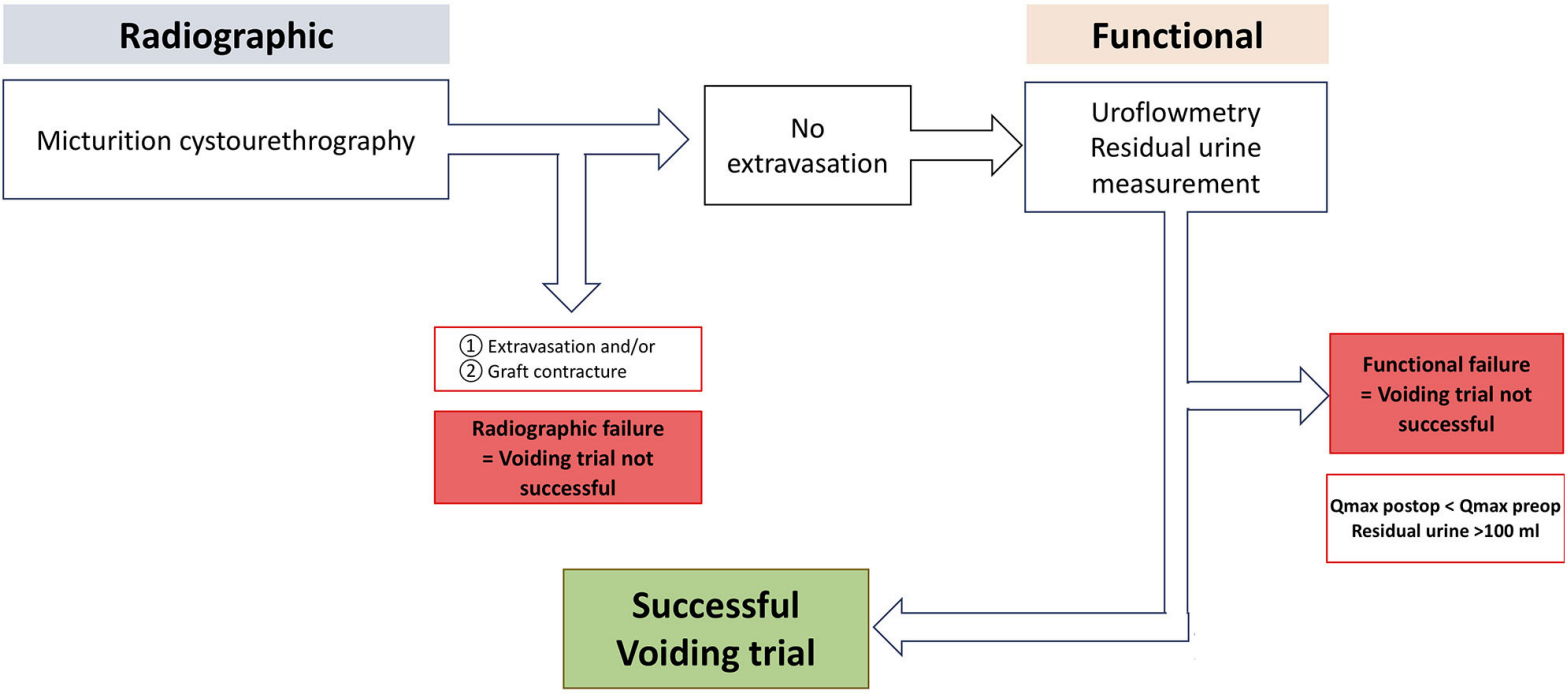
Institutional standardized radiological and functional voiding trial of patients after urethroplasty. Qmax, maximum urinary flow in uroflowmetry. With kind permission to use figure by M. W. Vetterlein (University Medical Center Hamburg-Eppendorf).

According to our institutional standardized algorithm (9, 12), all patients revisited our outpatient clinic 21 days after surgery to undergo radiographic and functional voiding trial (Figures 1, 3). Prior to any intervention urine analyses were performed. A voiding trial was deferred in case of a positive nitrite test on dipstick analysis or a non-treated positive urine culture. If no extravasation was investigated in MCU (radiographic success), functional voiding trial was performed by using uroflowmetry and sonographic residual urine measurement. In case of extravasation no further evaluation *via* uroflowmetry and post-void residual measurement was performed, catheterization was extended for further 7–14 days, depending on the extent of the extravasation and at the discretion of the surgeon, who performed the procedure. A successful voiding trial (Figure 3) was defined as an inconspicuous MCU and a residual urine volume of <100 mL (20).

### Clinical Follow Up, Definition of Stricture Recurrence and Urethral Stricture Surgery Patient-Reported Outcome Measure (USS-PROM)

All patients received a clinical follow-up undergoing physical examination, uroflowmetry and sonographic residual urine measure, 3 and 12 months postoperatively and annually thereafter. Stricture recurrence was defined as any required re-intervention (urethral dilation or endoscopic or surgical re-operation) (12). In addition, patients were evaluated with a validated USS-PROM (10, 11). It records functional results such as micturition (ICIQ-MLUTS short form, one question each of the ICSmale short form incontinence score and ICSQoL as well as Peeling's voiding picture), general condition (EQ-5D-3L), quality of life and patient satisfaction. The USS-PROM, which was validated and extended in German in 2013, also includes validated instruments for measuring continence (ICIQ-UI short form) and erectile function (IIEF erectile function domain) (1, 11).

### Statistical Analysis

Retrospective data collection and descriptive evaluation of patient characteristics was done (inclusion of all patients with voiding trial). For further analyses, all patients without available follow-up, with follow-up <3 months and/or pending second session were excluded. Categorical variables were calculated with frequencies and percentages, continuous variables with median and interquartile range (IQR). Significant differences in functional outcomes before and after intervention were assessed using the Wilcoxon test (significance level *p* < 0.05). Kaplan-Maier curves were generated to illustrate RFS.

## Results

### Patient Characteristics

**Figure 4:**
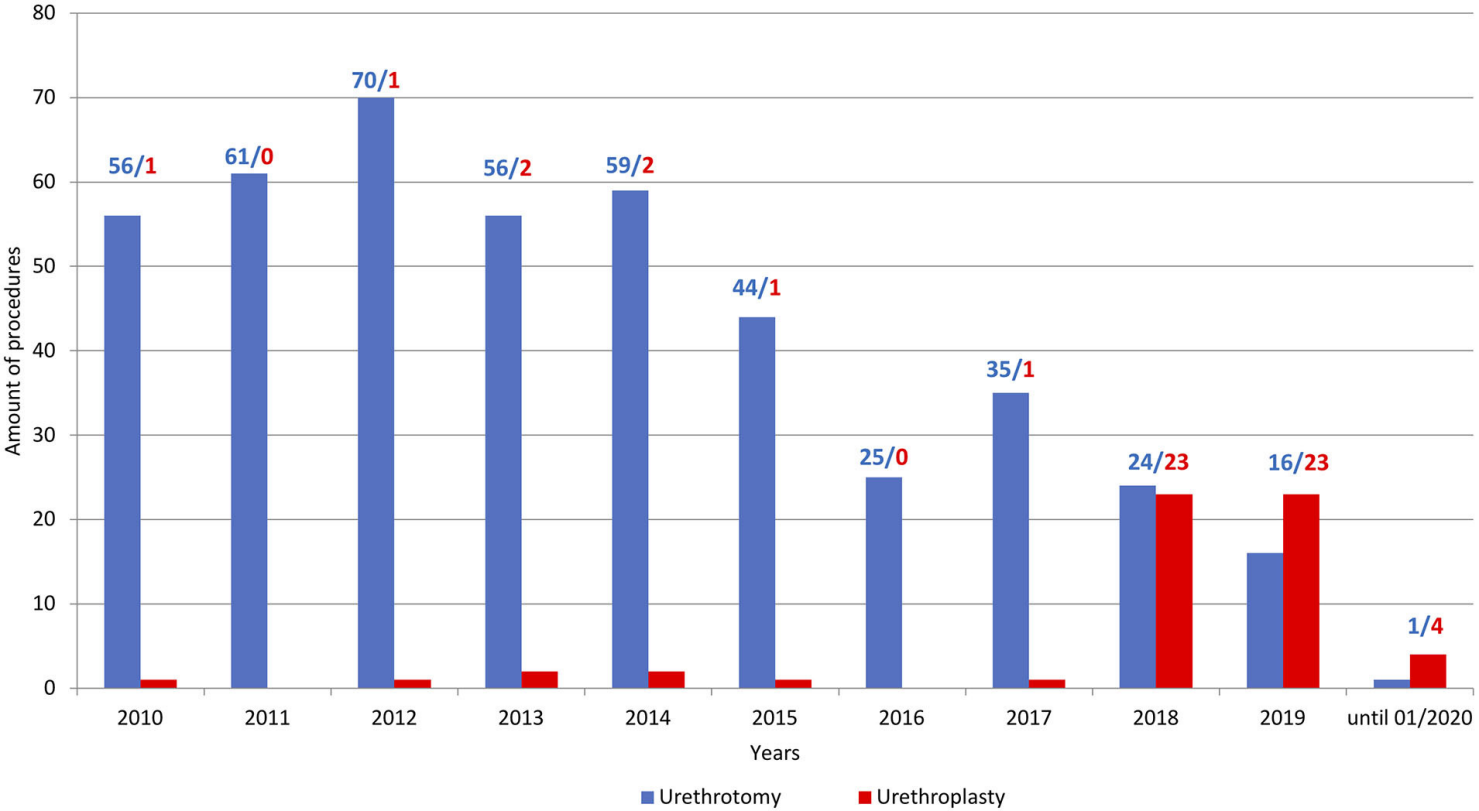
Barplots depicting the ratio of performed urethroplasties (red) vs. urethrotomies (blue) at the University Hospital Frankfurt between 2010 and January 2020.

Between January 2018 and January 2020, 50 patients underwent urethroplasty due to urethral stricture disease at our institution. In contrast to increasing procedures of urethroplasties, the number of urethrotomies decreased (Figure 4). Patient characteristics are summarized in Table 1. The median patient age was 57 (IQR 38–62) years. Of the total of 50 patients, 74% exhibited previous urethrotomy and 24% urethroplasty. Eighty-six percent of the patients received BMGU (median graft length 5.0 (IQR 4.5–7.0) cm, with 85% ventral onlay vs. 15% dorsal inlay technique. Other surgical procedures included excision and primary anastomoses (*n* = 1), bulboprostatic reanastomoses (*n* = 1) and preputial flaps (*n* = 5). Two-stage procedures were performed in 4 patients (8%) according to high complexity of urethral stricture. Peri- and postoperative complications occurred in a total of 5 (10%) patients: Three Clavien Dindo 3a (two abscesses and one bleeding from the oral graft donor site), one Clavien Dindo 2 (blood transfusion) and one Clavien Dindo 1 (suprapubic catheter dislocation) complications.

**Table 1:** Descriptive characteristics of 50 urethral stricture disease patients who received an urethroplasty at University Hospital Frankfurt between January 2018 and January 2020.

**Patient characteristics**	**Patients (*n* = 50)**
Age in years, media*n* (IQR)	57.0 (38–62)
ASA status *n* (%)	
I	15 (30.0)
II	26 (52.0)
III	9 (18.0)
BMI in kg/m^2^, media*n* (IQR)	25.0 (24.2–29.0)
Anticoagulation, *n* (%)	7 (14.0)
Diabetes mellitus, *n* (%)	7 (14.0)
Coronary heart disease, *n* (%)	2 (4.0)
Lichen sclerosus, *n* (%)	3 (6.0)
Previous pelvic radiation therapy, *n* (%)	2 (4.0)
Previous radical prostatectomy, *n* (%)	2 (4.0)
Previous TURP/HoLEP, *n* (%)	5 (10.6)
Previous Urethrotomies, *n* (%)	37 (74.0)
1	17 (34.0)
2	8 (16.0)
≥3	12 (24.0)
Previous Urethroplasty, *n* (%)	12 (24.0)
Previous hypospadia repair, *n* (%)	6 (12.0)
Previous meatus plastic, *n* (%)	3 (6.0)
Previous oral buccal mucosa graft, *n* (%)	3 (6.0)
Previous end to end anastomosis, *n* (%)	1 (2.0)
Localization of the stricture	
proximal membranous, *n* (%)	5 (10.0)
bulbar, *n* (%)	31 (62.0)
penobulbar, *n* (%)	3 (6.0)
Mid-penile, *n* (%)	5 (10.0)
Distal penile distal without including fossa navicularis/meatus urethrae, *n* (%)	3 (6.0)
Distal penile distal including fossa navicularis/meatus urethrae, *n* (%)	3 (6.0)

*IQR, interquartile range; BMI, body mass index; TURP, transurethral resection of the prostate; HoLEP, holmium enucleation of the prostate*.

### Radiographic and Functional Voiding Trial

Postoperative radiographic and functional voiding trial was successful in 85% of the patients on the 21. postoperative day. The maximum urinary flow (Qmax in median) in the uroflowmetry was significantly improved after postoperative voiding trial (24.0 vs. 7.0 mL/s; *p* < 0.01). Additionally, a significant reduction in the median residual urine volume was recorded (78 vs. 10 mL; *p* < 0.01).

### Clinical Follow Up, Definition of Stricture Recurrence and USS-PROM

**Figure 5:**
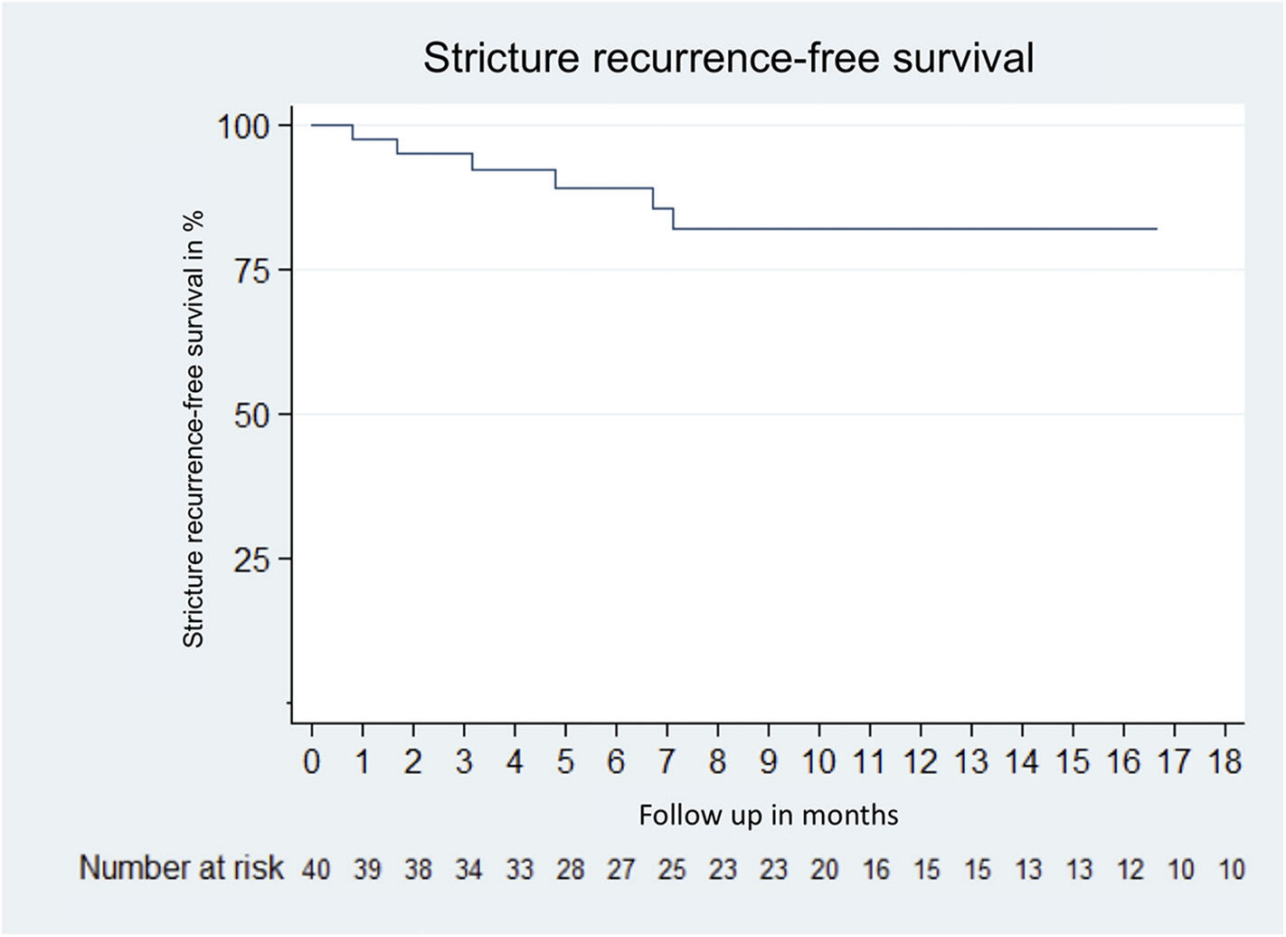
Kaplan–Meier curve of stricture recurrence-free survival of 40 patients who received urethroplasty due to urethral stricture disease between January 2018 and January 2020 at the University Hospital Frankfurt. Excluded patients were lost to follow up (*n* = 3); follow up <3 months (*n* = 8) and pending second stage procedure (*n* = 1).

In total, 50 patients underwent urethral surgery at UKF, after exclusion of patients lost to follow-up (*n* = 3), follow-up <3 months (*n* = 6) and outstanding second stage procedure (*n* = 1), 40 patients were eligible for final RFS analysis. Of these, 95% underwent our clinical follow-up. The USS-PROM response rate was 93%. With a median follow-up of 10 (IQR 5.1–18.0) months, the RFS was 83% (Figure 5).

Postoperatively, the evaluation of the USS-PROM showed a median ICS male VS of 4 (IQR 2–6, worst possible value 20) for micturition, an IIEF of 15 (IQR 5–27, best possible value 30) for erectile function, and an ICIQ of 0 (IQR 0–5, worst possible value 21) for incontinence. A total of 87% of the patients stated that they were not (54%) or only slightly (33%) burdened by the current micturition situation in daily life. According to patients who underwent BMGU, 41% reported mild to moderate persistent discomfort at the donor site in the mouth. These were manifested during eating (58%), speaking (25%) or a local numbness (67%). Overall, 95% of the patients stated that the quality of life had well-improved (62%) or slightly improved (33%) as a result of the performed urethral surgery. Additionally, 90% of patients were very satisfied (45%) or satisfied (45%) with the results of the surgery.

## Discussion

We demonstrated a successful implementation and transfer of an institutional standardized perioperative algorithm for urethral surgery from one location (UKE) to another (UKF). Based on comparable short-term FU with UKE (12), patients who underwent urethroplasty for urethral stricture disease showed excellent RFS, low complication rates, good functional results, improvement of QoL and high patient satisfaction. In a study period of 2 years, 50 complex urethral reconstructions were performed at the UKF. Conversely, in a survey of 845 (of 5771) German urologists, Rosenbaum et al. showed that only 8% of the respondents performed more than 5 urethroplasties per year and almost three quarters of the urologists do not perform this operation at all (21). Furthermore, a large-scale study by the Bertelsmann Foundation was able to show that clinics with greater specialization for a particular procedure are of higher quality and have fewer complications (22). In our short-term follow-up the RFS was 83%, which is in line with the current literature. In a large study of patients who underwent different urethroplasties for urethral stricture disease at the UKE, RFS was 88% at a similar median FU of 10 months (12). However, of those 205 included patients, only 68% (*n* = 140) were available for FU. The complication rate in our study was 10%, using Clavien-Dindo classification. These results are also comparable to the current literature, even though complication rates are rarely reported in urethral surgery. Spilotros et al. demonstrated a complication rate of 12.5% in a homogenous study of 128 BMGU patients. The most frequent complications were fistula formation, graft contracture, graft failure, postoperative bleeding from the oral graft donor site and wound infection (23).

Our standardized postoperative radiographic and functional voiding trial was successful in 85% of all patients. Vetterlein et al. recently demonstrated a comparably high successful voiding trial rate in a homogeneous cohort of BMGU patients (85%). In addition to standardization, the combined radiographic and functional voiding trial served as a risk stratification tool to identify those patients with an increased risk of early stricture recurrence (9).

The response rate of our USS-PROM was high with 93% in all patients eligible for follow-up. Interestingly, the patient-reported postoperative quality of life (95%) and patient satisfaction (90%) were higher than the surgical success rate (83%). These results are line with centers using the same USS-PROM (11, 24). In our study, a significant proportion of patients (67%) reported persistent numbness at the donor site in the mouth, a well-known morbidity side effect of BMGU (25). In a large prospective randomized study evaluating outcome of closure vs. non-closure of buccal mucosa harvesting site in 135 BMGU patients, Soave et al. demonstrated that 71% still reported numbness 6 months after surgery, 17% of those strongly (26). PROMs are essential for the clinical follow-up as they allow for an objective outcome evaluation and comparison of results between different centers, if similar PROMs were provided (24, 27, 28). Additional quality of life and patient satisfaction assessment may serve as a surrogate for the overall success of urethral surgery.

Limitations of our study was firstly the retrospective study design. Second, no baseline evaluations with the USS-PROM before the urethroplasties were available for comparisons. Furthermore, the follow-up was still relatively short. However, when comparing RFS and overall patient satisfaction with the UKE in a similar observation period, our results are comparable (12).

## Data Availability Statement

The raw data supporting the conclusions of this article will be made available by the authors, without undue reservation.

## Ethics Statement

The studies involving human participants were reviewed and approved by Ethic committee, University Hospital Frankfurt, 82/19. Written informed consent for participation was not required for this study in accordance with the national legislation and the institutional requirements.

## Author Contributions

MW: manuscript writing/editing, protocol/project development, data collection or management, and data analysis. MK: data analysis and protocol/project development. BL and BH: data collection or management. MM and FK-HC: manuscript writing/editing and data collection or management. PM: data analysis and data collection or management. AB: manuscript writing/editing. MV and SM: manuscript writing/editing and protocol/project development. RD, MF, and LK: manuscript writing/editing, protocol/project development and data analysis. All authors contributed to the article and approved the submitted version.

## Conflict of Interest

The authors declare that the research was conducted in the absence of any commercial or financial relationships that could be construed as a potential conflict of interest. The reviewer SAh declared a past co-authorship with several of the authors RD, MV, MF, FK-HC, AB, and LK to the handling editor.
